# RNAscope in situ hybridization reveals microvascular sequestration of *Plasmodium relictum* pSGS1 blood stages but absence of exo-erythrocytic dormant stages during latent infection of *Serinus canaria*

**DOI:** 10.1186/s12936-024-04899-x

**Published:** 2024-03-08

**Authors:** Tanja Himmel, Josef Harl, Julia Matt, Nora Nedorost, Tatjana Iezhova, Mikas Ilgūnas, Gediminas Valkiūnas, Herbert Weissenböck

**Affiliations:** 1 Institute of Pathology, Department of Pathobiology, University of Veterinary Medicine Vienna, Veterinaerplatz 1, 1210 Vienna, Austria; 2 Nature Research Centre, Akademijos 2, 08412 Vilnius, Lithuania

**Keywords:** Parasitaemia, Relapse, Recrudescence, Dormant stages, Exo-erythrocytic stages, Erythrocytic merogony, Phanerozoites

## Abstract

**Background:**

Birds chronically infected with avian malaria parasites often show relapses of parasitaemia after latent stages marked by absence of parasites in the peripheral circulation. These relapses are assumed to result from the activation of dormant exo-erythrocytic stages produced during secondary (post-erythrocytic) merogony of avian *Plasmodium* spp. Yet, there is no morphological proof of persistent or dormant tissue stages in the avian host during latent infections. This study investigated persistence of *Plasmodium relictum* pSGS1 in birds with latent infections during winter, with the goal to detect presumed persisting tissue stages using a highly sensitive RNAscope® in situ hybridization technology.

**Methods:**

Fourteen domestic canaries were infected with *P. relictum* pSGS1 by blood-inoculation in spring, and blood films examined during the first 4 months post infection, and during winter and spring of the following year. After parasitaemia was no longer detectable, half of the birds were dissected, and tissue samples investigated for persisting tissue stages using RNAscope ISH and histology. The remaining birds were blood-checked and dissected after re-appearance of parasitaemia, and their tissues equally examined.

**Results:**

Systematic examination of tissues showed no exo-erythrocytic stages in birds exhibiting latent infections by blood-film microscopy, indicating absence of dormant tissue stages in *P. relictum* pSGS1-infected canaries. Instead, RNAscope ISH revealed rare *P. relictum* blood stages in capillaries of various tissues and organs, demonstrating persistence of the parasites in the microvasculature. Birds examined after re-appearance of parasitemia showed higher numbers of *P. relictum* blood stages in both capillaries and larger blood vessels, indicating replication during early spring and re-appearance in the peripheral circulation.

**Conclusions:**

The findings suggest that persistence of *P. relictum* pSGS1 during latent infection is mediated by continuous low-level erythrocytic merogony and possibly tissue sequestration of infected blood cells. Re-appearance of parasitaemia in spring seems to result from increased erythrocytic merogony, therefore representing recrudescence and not relapse in blood-inoculated canaries. Further, the study highlights strengths and limitations of the RNAscope ISH technology for the detection of rare parasite stages in tissues, providing directions for future research on persistence and tissue sequestration of avian malaria and related haemosporidian parasites.

**Supplementary Information:**

The online version contains supplementary material available at 10.1186/s12936-024-04899-x.

## Background

Parasites of the genus *Plasmodium* (Haemosporida, Apicomplexa) infect birds of most orders all over the world, except Antarctica [1]. Some *Plasmodium* species cause severe avian malaria, which can be fatal in non-immune hosts due to acute high parasitaemia and damage of internal organs caused by excessive tissue merogony of the parasites [2–6]. However, in birds sharing co-evolutionary history with the parasites, *Plasmodium* infections tend to be mild, although adverse consequences on fitness, reproduction, and survival have been reported [7–10].

Birds sampled in the wild typically show subclinical infections characterized by low parasitaemia intensities (often below 0.01%) [11]. These are usually chronic infections enduring in individuals that have survived the acute primary infection. There is evidence that malaria and other haemosporidian parasites can persist in the avian host for many months or even years and fluctuations in parasitaemia intensity are usually observed in chronically infected birds [12–18]. Often, the parasites disappear from the peripheral circulation or drop to intensities that may not be detectable in blood films (latent or subpatent infection) but re-occur after longer periods of latency without re-infection by mosquitoes [11, 15, 19–21]. Such sudden re-appearances of parasitaemia (often referred to as ‘relapses’) may occur at irregular intervals after the primary infection but are typically observed during spring in naturally infected birds of temperate regions [11, 16, 22–27]. Experimental studies have identified some environmental and physiological factors triggering recurrent parasitaemia in birds, including seasonal changes in the photoperiod [18, 19, 24], elevated corticosterone levels [23] and stress [28], as well as exposure to mosquito bites [13]. However, the source of parasitaemia ‘relapses’, i. e. the parasite stages causing them, remain largely unknown, hampering our understanding of persistence mechanisms in avian malaria and related haemosporidioses.

In primate malaria, relapses (re-appearance of parasites in the blood after a period of absence) have been linked to the presence of dormant stages (hypnozoites) in hepatocytes of the liver, the main site of exo-erythrocytic merogony of mammalian *Plasmodium* species [29]. Strictly speaking, only re-appearance of parasitaemia originating from hypnozoites are considered true relapses, while re-appearance of parasites involving increased parasitaemia due to multiplication of blood stages is called recrudescence [1]. The term ‘hypnozoite’, derived from the Greek (*hypnos* – sleep, *zoon* – animal) and originally coined by Markus [30], was adopted by Garnham and other researchers to describe the latent nature of this parasite stage associated with subclinical infections [29, 31]. Morphological evidence for hypnozoites in *Plasmodium* species first came from studies of the simian parasite *Plasmodium cynomolgi* and consisted of small intracytoplasmic, uninucleated parasite forms detected in liver cells of rhesus monkeys months after sporozoite inoculation [32, 33]. Later, hypnozoites were demonstrated by immunofluorescence assays in another simian parasite, *Plasmodium simiovale* [34], as well as in the human malaria species *Plasmodium vivax* [33, 35, 36]*,* and consequently served to explain periodic increases in parasitaemia observed during some *Plasmodium* infections in humans. Common theory today is that hypnozoites are sporozoite-derived uninucleated trophozoites which persist as dormant forms in hepatocytes and, upon activation, produce hepatic meronts whose merozoites initiate the development of erythrocytic stages [37–39]. Recently, in vitro activation of hypnozoites of the simian parasites *P. cynomolgy* was shown to induce liver stage maturation and merozoite formation, supporting the idea that hypnozoites are the cause of relapses [40]. While it is still unclear, whether all relapses observed during simian and human malaria result from the activation of hypnozoites, the concept of dormant stages persisting in the vertebrate host is generally accepted [41].

Likewise, in avian malaria and other related bird haemosporidian parasites, dormant stages have been postulated for a long time, but not yet demonstrated convincingly. It is assumed, that parasitaemia relapses during avian malaria might be related to the activation of “sleeping” exo-erythrocytic meronts produced during secondary (post-erythrocytic) merogony, so-called phanerozoites [11, 14]. In contrast to human and simian malaria, avian *Plasmodium* merozoites released from erythrocytic meronts may re-invade tissue cells (mostly endothelial cells) and initiate secondary exo-erythrocytic merogony [5, 14]. The hypothesis, that phanerozoites might contribute to rises in parasitaemia after periods of latency is supported by the fact that relapses are observed in birds inoculated with *Plasmodium*-infected blood [16, 25] rather than sporozoites [42]. However, patterns of persistence of avian malaria and other haemosporidian parasites remain unclear with no morphological proof of persistent stages in the avian host analogous to reported hypnozoites of some primate malaria species. The main reason for missing evidence of persisting stages in avian malaria and related haemosporidian parasites is related to detection difficulties and the lack of targeted research. Provided that persisting stages are dormant, it can be assumed that they are small and scarce like primate malaria hypnozoites and hence evasive for conventional microscopic techniques. Current in situ hybridization methods, which generally enable the specific identification of exo-erythrocytic stages of avian haemosporidians [43, 44] have not yet been able to detect tissue stages in birds with PCR-proven latent infections [45] or long-lasting parasitaemia [46].

The present study aimed to address this issue by an experimental approach using *Plasmodium relictum* (lineage pSGS1) and domestic canaries *Serinus canaria* as parasite-host system. *Plasmodium relictum* pSGS1 is a cosmopolitan parasite with a broad range of avian hosts and is a convenient candidate to tackle the question about dormant stages in avian malaria parasites because it is known to cause long-lasting chronic infections marked by relapses [11, 42, 45]. The objective of this study was to investigate tissues of infected canaries during the latent period of infection during winter with the goal to detect presumed persisting tissue stages using a highly sensitive RNAscope^®^ in situ hybridization (ISH) technology.

## Methods

### Experimental design

The infection experiment was carried out at the Nature Research Center (NRC), Vilnius, Lithuania from 2021–2022. For the experimental infections, 7-months-old domestic canaries *Serinus canaria* were purchased commercially from licensed breeders at Vogelhandel Van der Wegen (Steenbergen, Netherlands). The birds were kept in individual cages in a vector-free room under controlled conditions (20 ± 1 °C; 50–60% relative humidity) and exposed to a natural photoperiod with light/dark cycles as for the location of Vilnius. Prior to the sub-inoculation experiments, all recipient birds were proven to be uninfected with haemosporidian parasites by microscopic examination of blood films and by negative PCR-based testing of blood samples (see "Molecular analysis" section).

**Fig. 1:**
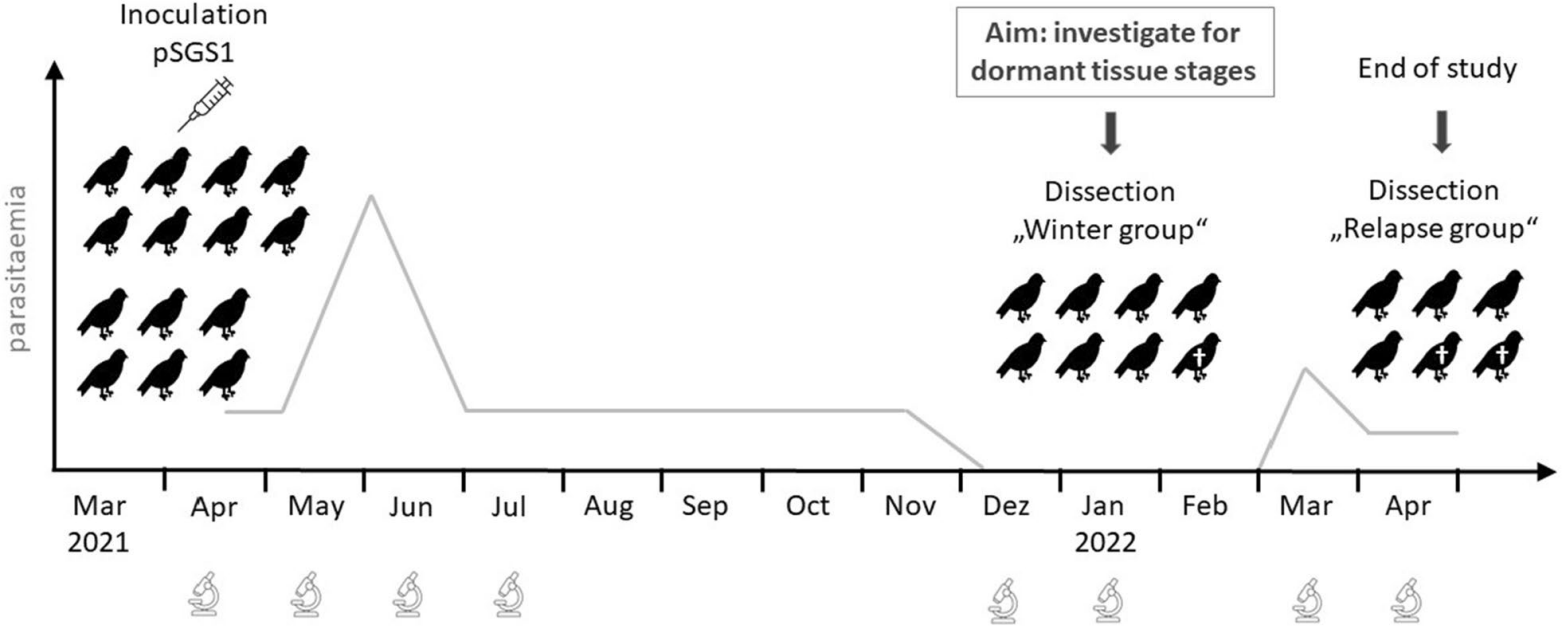
Study design schematic. Domestic canaries *Serinus canaria* (*n* = 14) were inoculated with *Plasmodium relictum* pSGS1 in March/April 2021. Blood samples were taken, and blood films continuously examined within the first 4 months post infection, and once in December and January, respectively. After parasitaemia was no longer detectable by microscopic examination during winter (January 2022), seven birds from the ‘winter group’ (one died naturally—indicated by obelisk) were dissected and tissue samples investigated for dormant stages. The remaining six birds (‘relapse group’) were continuously blood-checked every 3–4 days during spring (March, April) of the following year to check for re-appearance of parasitaemia. While two birds died before relapse, the other four birds were euthanized and dissected after re-appearance of parasitaemia (relapse or recrudescence) in April 2022. An uninfected control group of six birds was investigated in parallel, six of which were dissected in January 2022 and six in April 2022 (not shown in the schematic)

A cryo-preserved strain of *P. relictum* (lineage pSGS1) was used to initiate experimental infections of 14 canaries in March 2021. First, a cryopreserved sample was used to multiply the strain in two donor canaries *S. canaria*. Briefly, the frozen tube containing infected blood was thawed and mixed with 12% NaCl (one-third of the thawed sample amount). After equilibration for 5 min at room temperature, one volume of 1.6% NaCl was added, followed by centrifugation at 200 g for 5 min. After centrifugation, the supernatant was removed and 1.6% NaCl (one-third of the original sample) was added and centrifuged again. After removing the supernatant, the same procedure was repeated three times with 0.9% NaCl solution. The final mixture was diluted with 0.9% NaCl and sub-inoculated into the recipient birds, as described by Palinauskas et al*.* [47]. After successful development of parasitaemia in the donor birds, their infected blood was used to inoculate the 14 experimental *S. canaria*. Success of the infections was checked by examination of blood films prepared from blood samples taken from the birds between 6 and 24 days post infection (dpi). Within the first 4 months after inoculation, parasitaemia was continuously monitored every 1–2 weeks. Thereafter, parasitaemia was checked once in December 2021 and January 2022, respectively. When parasitaemia was no longer detectable in the birds during winter based on blood film examinations (latent infection), seven of the inoculated birds and three uninfected control birds (in the following referred to as “winter group”) were euthanized and dissected for investigation of dormant tissue stages. One bird died naturally on July 13, 2021. The remaining birds (referred to as “relapse group”) were maintained in the laboratory and served to check for recurrence of parasitaemia by blood film examinations. Two birds of the relapse group died naturally before recurrence of parasitaemia in March and they were dissected. All other exposed birds were euthanized after relapse and dissected along with the three remaining control birds (Fig. 1).

### Microscopic examination of blood films

Blood samples were obtained by puncturing the brachial vein using a sterile needle and collecting blood into a heparinized microcapillary. Drops of blood were used to prepare each two blood films immediately after withdrawal, and the blood films were air-dried, fixed by dipping the slides in absolute methanol, and stained with 10% Giemsa solution. Blood films were microscopically examined using an Olympus BX61 (Olympus, Tokyo, Japan) light microscope equipped with a DP70 digital camera and the imaging software AnalySIS FIVE (Olympus) following a standard protocol for the detection of avian haemosporidian parasites [11].

### Collection of tissue samples

For histology, the following tissues were collected during bird dissections: brain, heart, lungs, trachea, liver, spleen, oesophagus, proventriculus, gizzard, intestines, kidneys, ovaries, testicles, pectoral muscle, bone marrow, and skin from the forehead and orbital region. Tissue samples were fixed in 10% neutral buffered formalin for 24 h at room temperature. After fixation, the samples were washed in distilled water for 15 min, dehydrated in an increasing series of 70–100% ethanol, and clarified in xylene before embedding in paraffin wax. Additionally, tissue samples of the liver, lungs, and brain of the birds dissected during winter were fixed in 96% ethanol for molecular analysis.

### Molecular analysis

In addition to microscopic examinations of blood films, tissue samples from birds dissected during winter were molecularly screened for *Plasmodium* parasites. DNA was extracted from ethanol-fixed tissue samples using the DNeasy Blood & Tissue Kit (Qiagen, Venlo, Netherlands) and following the manufacturer’s instructions except for one modification; the DNA was eluted twice with each 100 µl of AE buffer, and the second eluate was used as PCR template.

PCR was performed applying a nested PCR protocol targeting the 478 bp haemosporidian barcode region of the mitochondrial cytochrome b gene (*cytb*) gene of the parasites [48]. First, the primer pair HaemNFI/HaemNR3 was used to amplify DNA of *Plasmodium*, *Haemoproteus*, and *Leucocytozoon*. Using 1 µl of amplicon from the first PCR product, the primer pair HaemF/HaemR2 was used in the nested PCR to amplify DNA of *Plasmodium*/*Haemoproteus*.

PCRs were performed in 25 µl reaction volumes containing 14.375 µl of nuclease-free water, 5 µl of 5X Green GoTaq Flexi Buffer (Promega, Madison, Wisconsin, USA), 2 µl of MgCl2 solution (25 mM), 0.5 µl of PCR nucleotide mix (10 mM, Promega), 0.125 µl of GoTaq G2 Flexi DNA Polymerase (5 u/µl, Promega), 1 µl each of forward and reverse primers (10 pmol/µl), and 1 µl of DNA template. PCR reactions were run with an initial step of 2 min at 94 °C, followed by 35 cycles of 30 s at 94 °C, 30 s at 50 °C, and 1 min at 72 °C, followed by a final extension step of 10 min at 72 °C. PCR amplifications were evaluated by gel electrophoresis of 5 µl of nested PCR products run on 1% agarose gels stained with Midori Green Advance (Nippon Genetics Europe, Dueren, Germany). Amplicons were visualized with a BioSens SC-Series 710 gel documentation system (GenXpress, Wiener Neudorf, Austria). In every PCR run, a positive (*Plasmodium*-positive sample) and a negative (nuclease-free water) control were included.

### Histological examination

For histological examinations, sections of 2–3 µm were cut from all formalin-fixed paraffin-embedded (FFPE) tissue samples. Of every bird and organ, each one section was prepared and processed following standard histological techniques (see [49]). After mounting of sections on glass slides, they were air-dried for 30 min at 50 °C. Sections were deparaffinized with limonene (SAV Liquid Production, Flintsbach, Germany) and rehydrated with 100%, 96% and 70% ethanol, and distilled water. Then, sections were stained with haematoxylin and eosin (HE), cover-slipped, and microscopically screened for exo-erythrocytic parasite stages at 100x, 200x, and 400 × magnifications using an Olympus B51 light microscope equipped with an Olympus UC90 digital camera (Olympus, Tokyo, Japan).

### In situ hybridizations

To detect *Plasmodium* blood and tissue stages, each one section of all organs from every bird were subjected to conventional chromogenic ISH using previously established protocols [50, 51]. In addition, each one section of all organs were investigated applying the highly sensitive RNAscope^®^ ISH technology, which allows visualization of single RNA molecules in FFPE tissues using a hybridization-based signal amplification system [52].

### Chromogenic ISH

For chromogenic ISH, sections of 2–3 µm were cut and mounted on SuperFrost Plus Adhesion slides (Epredia, Fisher Scientific, Vienna, Austria). Chromogenic ISH was carried out using a *Plasmodium* genus-specific probe (Plasmo18S) following previous protocols [43, 53]. In brief, FFPE tissue sections were deparaffinized, rehydrated, and subjected to proteolytic treatment using proteinase K (#03115828001, Merck, Darmstadt, Germany) 3 µg/ml in tris-buffered saline at 37 °C for 40 min. After proteolysis, sections were washed in distilled water, dehydrated in 96% and 100% ethanol, and air-dried. Then, sections were placed in a humid chamber, covered with hybridization solution containing 1 ng/100 µl of digoxigenin-labelled probe and incubated overnight at 40 °C. On the next day, sections were subjected to stringency washes in saline-sodium citrate (SSC) buffer (pH 7.0) and incubated with normal goat serum blocking reagent (VESCS-1000, Szabo-Scandic, Vienna, Austria) containing 10% Triton X-100 (#648466, VWR International, Vienna, Austria) for 30 min. To detect the digoxigenin-labelled hybrids, anti-digoxigenin-AP Fab fragments (#11093274910, Merck) were applied to the sections at a concentration of 1:200. To visualize the parasite-probe hybrids, a chromogenic reaction was performed using the colour substrates 5-bromo-4-chloro- 3-indolyl phosphate (BCIP) (#11383221001, Merck) and 4-nitro blue tetrazolium chloride (NBT) (#11383213001, Merck). After stopping the chromogenic reaction with tris–EDTA buffer (#1.08382 and #1.12029, Merck), sections were counterstained with haematoxylin (#105174, Merck) and mounted using Aquatex (#1.08562, Merck) and coverslips. For every ISH procedure, a tissue sample previously confirmed positive for *Plasmodium* sp. was included as positive control.

### RNAscope^®^ ISH

In parallel to chromogenic ISH, tissue samples of all birds (except the bird which died naturally in July 2021) were tested for *Plasmodium* parasite stages applying the commercially available RNAscope^®^ ISH technology. In contrast to standard chromogenic ISH, RNAscope provides higher sensitivity due to a hybridization-based signal amplification system. For this purpose, a custom *P. relictum* pSGS1-specific oligonucleotide probe (consisting of two double “Z” probe pairs), was designed by Advanced Cell Diagnostics (Advanced Cell Diagnostics, Newark, CA, USA) based on a previously published 18S rDNA sequence of *P. relictum* [54]. The designed RNAscope ZZ probes (RNAscope^®^ Probe B-P. relictum-18SrRNA-O1-C1, catalog #1215001-C1) are complementary to nucleotide positions 127–168 (5'-GCT TAA CAA ATA CGT GTT CTA CAG AAC CTT TTT GGG GAA CTG-3') and 1753–1800 (5'-GAT AAA GAT TAC CTA CAC TTT TCA GTG GAG GAA AAT TAT ACC TTT TGT-3') of the *18S* rDNA sequence of clone *P. relictum* pSGS1 (Genbank accession no. MK650474). RNAscope^®^ ISH was performed using 2–3 µm tissue sections mounted on SuperFrost Plus Adhesion slides (Epredia) and applying the chromogenic RNAscope^®^ 2.5 HD Reagent Kit-RED assay (catalog #322350) following the manufacturer’s protocol.

Prior to processing the samples with RNAscope^®^ ISH, the probe and assay were tested on a tissue sample previously confirmed positive for *P. relictum* tissue stages by chromogenic ISH (positive control). To check probe specificity, a tissue sample from an uninfected bird was used as negative control. Background staining related to the assay was evaluated by omitting the probe on a sample positive for *P. relictum* pSGS1 tissue stages, and by applying the dihydrodipicolinate reductase (dapB), a bacterial gene negative control probe (ACD, catalog #200470) on a sample positive for *P. relictum* pSGS1 as confirmed previously. In each RNAscope^®^ ISH assay, the positive control was included.

All H&E-stained and ISH-processed slides were examined at low (100x, 200x) and high (400x, 1000x) magnifications using an Olympus B51 light microscope equipped with an Olympus UC90 digital camera (Olympus). Photographs were acquired using the image software cellSens Entry (Olympus) and assembled in Adobe Photoshop CC 2023 (Adobe, San José, CA, USA), including adjustments for brightness and contrast.

## Results

### Parasitaemia dynamics

All inoculated birds (*n* = 14) developed parasitaemia within 24 dpi (*m* = 13) (Table 1). Peak of parasitaemia was recorded between 10–24 dpi (*m* = 16), with peak intensities ranging from 0.03 to 6% (*m* = 1.02). After the peak, the intensity decreased in all birds and ranged between 0.003 and 0.2% in the following 2 months. One infected bird (AH2181) died naturally 84 dpi (July 13, 2021). Parasitaemia in this bird was detected 6 dpi and reached its peak of 3.0% 13 dpi. After the peak, parasitaemia decreased again and stayed ≤ 0.02% in this individual. At the final two checks before death (56 and 76 dpi), the bird showed no parasitaemia. All other infected birds survived until the following year. During blood film examinations in December, parasitaemia was still detectable in all infected birds. Single erythrocytic meronts were seen in two birds at this time (AH2213 and AH2204). Latency, i.e., no detectable parasitaemia by blood film examination, was recorded in most birds in January 2022 after 264–299 dpi (*m* = 270), except for one individual (AH2217) which showed a parasitaemia of 0.005% one day before euthanization of all birds from the winter group on January 13. This bird showed latency only in mid-March. Inoculated birds from the relapse group maintained latent infections until early spring (mid-March). Re-appearance of parasites in the blood was observed in four individuals 332–367 dpi, indicating relapse, but parasitaemia stayed relatively low ranging between 0.005 and 0.3%. During relapse, blood stages (trophozoites, meronts, gametocytes) were readily detectable by blood film examination in these birds. Two birds of the relapse group (AH2213, AH2214) were found dead 318 dpi (March 4, 2022) and 334 dpi (March 16, 2022), respectively, before blood film examination in spring. During dissection of the latter bird, a small amount of blood was taken from the heart and a blood film prepared which showed plenty infected erythrocytes infected with *Plasmodium*, indicating relapse and high parasitaemia at time of death. All other birds from the relapse group were euthanized on April 27, 2022, and dissected. Uninfected control birds remained negative for *Plasmodium* parasites during the entire study.

**Table 1:** Parasitaemia intensities, latency, and relapse (re-appearance of parasitaemia) recorded by blood film examinations over the course of experimental *Plasmodium relictum* pSGS1 infections in domestic canaries *Serinus canaria* (*n* = 14)

Bird ID	Group	Infection date	1st Parasitaemia (dpi)	Peak (dpi)	Chronic phase (May-Jul 2021)	1st Winter check (10–12-21)	2nd Winter check (12–01-22)	Dissection date winter group	Relapse (dpi)	Spring parasitaemia (Mar-Apr 2022)	Dissection date relapse group
AH2181	-^a^	20-04-21	0.006 (6)	3.0 (13)	0–0.3 (0.047)	N/A^a^	N/A^a^	13-07-21	–	–	–
AH2203	W	20-04-21	0.004 (6)	0.03 (13)	0–0.02 (0.012)	0.01	0	13-01-22	–	–	–
AH2204	W	23-04-21	0.26 (10)	6.0 (17)	0–0.06 (0.014)	2.2	0	13-01-22	–	–	–
AH2205	W	20-04-21	0.1 (6)	0.4 (13)	0–0.08 (0.033)	0.005	0	13-01-22	–	–	–
AH2206	W	16-04-21	0.03 (10)	0.9 (24)	0–0.1 (0.040)	0.02	0	13-01-22	–	–	–
AH2207	W	16-04-21	0.1 (10)	0.2 (24)	0–0.04 (0.011)	0.01	0	13-01-22	–	–	–
AH2208	W	19-03-21	0.04 (10)	1.0 (18)	0–0.1 (0.016)	0.002	0	13-01-22	–	–	–
AH2209	W	23-04-21	0.24 (10)	2.1 (17)	0–0.06 (0.015)	0.005	0	13-01-22	–	–	–
AH2213	R	20-04-21	0.1 (13)	0.1 (13)	0–0.08 (0.023)	0.1	0	–	N/A^b^	N/A^b^	04-03-22
AH2214	R	16-04-21	0.1 (10)	0.1 (10)	0–0.05 (0.022)	0.02	0	–	N/A^b^	N/A^b^	16-03-22
AH2215	R	16-04-21	0.4 (10)	0.4 (10)	0–0.2 (0.051)	0.02	0	–	0.04 (335)	0.03–0.3 (0.081)	27-04-22
AH2216	R	23-04-21	0.67 (10)	0.67 (10)	0–0.01 (0.005)	0.02	0	–	0.007 (332)	0.007–0.06 (0.030)	27-04-22
AH2217	R	19-03-21	0.09 (18)	0.3 (24)	0.003–0.03 (0.009)	0.01	0.005	–	0.007 (367)	0.005–0.03 (0.012)	27-04-22
AH2218	R	16-04-21	0.1 (24)	0.1 (24)	0.02–0.08 (0.038)	0.02	0	–	0.05 (335)	0.02–0.2 (0.094)	27-04-22

*W* winter group, *R* relapse group, *dpi* days post infection, *N/A* not available

^a^Bird AH2181 was not assigned to a group because it died early during the experiment (13-07-2021)

^b^Data on relapse and spring parasitaemia are not available due to death of birds before blood film examinations

### Molecular results “winter group”

PCR screening of blood samples obtained from the birds dissected during winter (latency) showed positive results for two of seven birds (AH2204 and AH2206); other birds were negative, corresponding to negative results from the blood film screenings and confirming latency in most birds. By contrast, PCR screening of tissue samples obtained during dissection of the same birds revealed positive results in all cases, indicating that the *Plasmodium* infections were not entirely cleared by the hosts’ immune response at this point of investigation. The uninfected control birds of the winter group were PCR-negative.

### Histological examinations

Histological examinations revealed no exo-erythrocytic meronts in the organs of the birds, neither from the winter group, nor from the relapse group. Two infected birds from the winter group (AH2204 and AH2207), showed severe enlargement of the spleen during macroscopic examination. Occasionally, mild to severe infiltrations of mononuclear inflammatory cells were seen in the liver and lungs of both *Plasmodium*-infected and uninfected control birds from the winter and relapse group, respectively.

### RNAscope and chromogenic ISH results

**Fig. 2:**
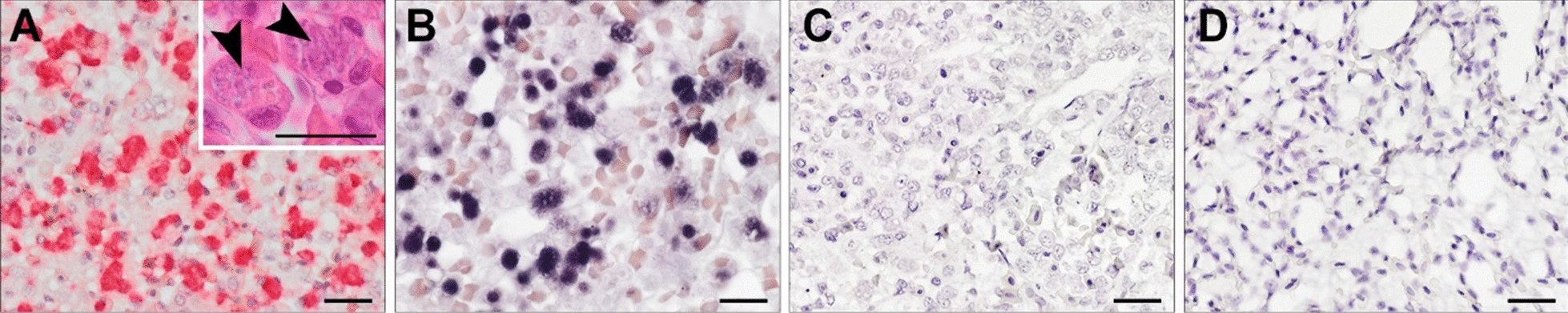
Results of testing the RNAscope in situ hybridization (ISH) assay for detecting *Plasmodium relictum* in tissue sections. **A** RNAscope ISH showed bright red labelling of *P. relictum* stages in a lung section from an infected penguin (positive control) displaying numerous blood and tissue meronts (arrowheads) by HE-staining (insert). **B** A similar staining pattern was observed in the positive control with chromogenic ISH applying a *Plasmodium* genus-specific probe. **C**, **D** Specificity of the RNAscope assay was confirmed by negative ISH result when applying the RNAscope probe on a sample from an uninfected control bird (**C**) and by applying a negative control probe (DapB) on a positive control sample (**D**). Scales = 20 µm

Testing of the RNAscope ISH assay on the positive control sample showed bright red staining of *P. relictum* parasite stages (Fig. 2A). This positive control sample obtained from a penguin was previously confirmed positive for *P. relictum* by chromogenic ISH using a *Plasmodium*-specific probe (Fig. 2B). Specific binding of the RNAscope probe to *P. relictum* stages was confirmed by negative ISH result when applying the probe on a tissue section from an uninfected control bird (Fig. 2C). Application of the negative control probe on a *P. relictum*-positive sample also resulted in negative outcome, confirming the specificity of the RNAscope assay (Fig. 2D).

In some tissues, for example the kidneys, little to moderate unspecific background staining was observed (Additional file 1A), which could be related to the assay rather than to unspecific binding of the probe, as the same staining pattern was also observed when the RNAscope probe was omitted during ISH (Additional file 1B).

**Fig. 3:**
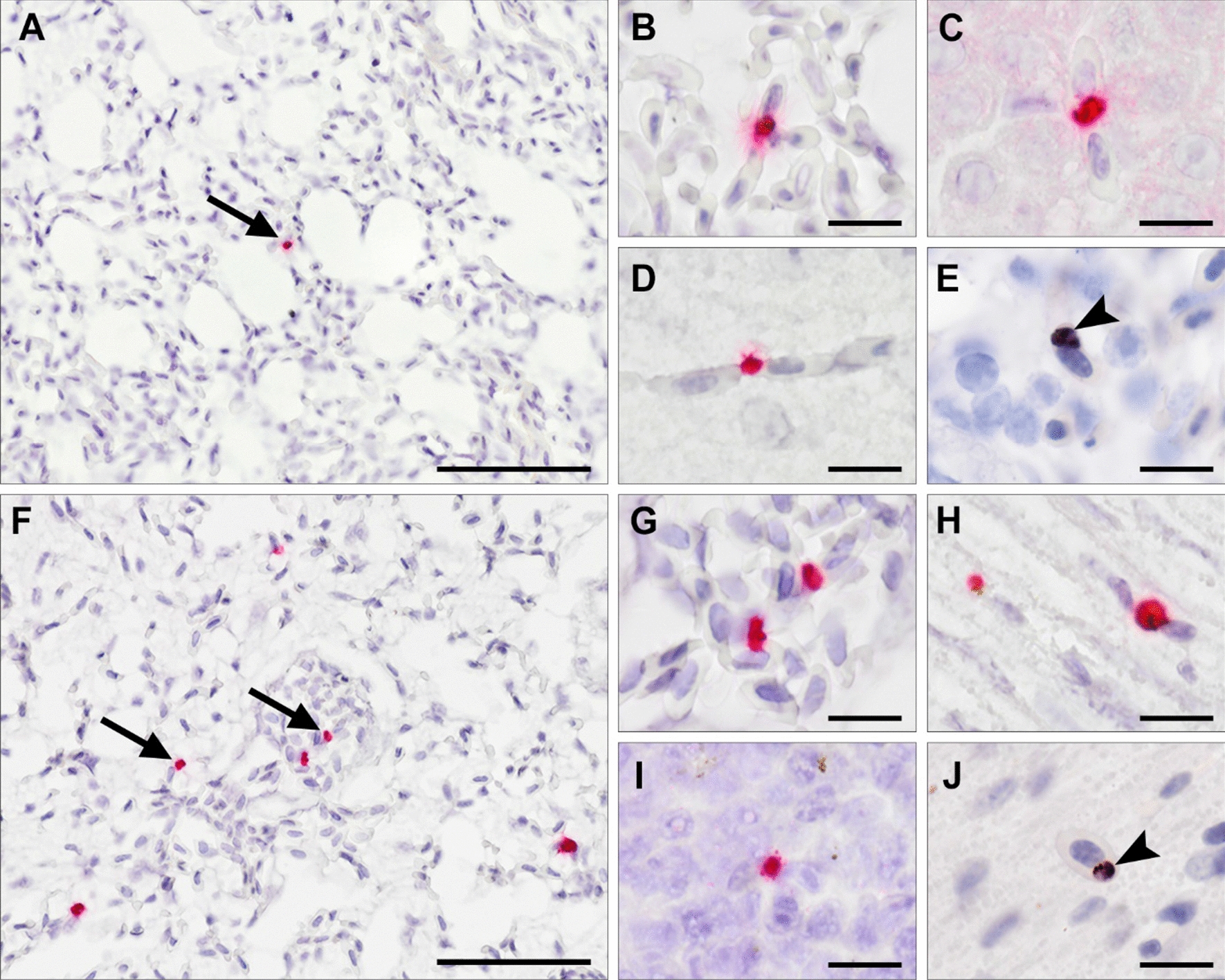
*Plasmodium relictum* pSGS1 blood stages detected during latency (**A**–**E**) and after re-appearance of parasitaemia (**F**–**J**) in experimentally infected *Serinus canaria* using RNAscope (**A**–**D**, **F**–**I**) and chromogenic (**E**, **J**) in situ hybridization (ISH). **A**–**E** Birds dissected during winter (latency) showing rare blood stage signals (arrows) in capillaries of the lung (**A**, **B**, **E**), liver (**C**), and brain (**D**). RNAscope signals were comparable to chromogenic ISH blood stage signals located in infected erythrocytes (**E**, arrowhead). No exo-erythrocytic stages were found in these birds. **F**–**J** Birds dissected after relapse showing plenty blood stage signals (arrows) in capillaries and larger blood vessels of the lung (**F**, **G**), heart muscle (**H**, **J**) and spleen (**I**). Scale bars = 50 µm (**A**, **F**) and 10 µm (**B**–**E**, **G**–**J**)

Applying the RNAscope probe on the tissue samples from the experimental birds showed few bright red signals in different organs of six of seven infected birds dissected during winter (Table 2). The signals appeared small and roundish (~ 3 µm in size), and were mostly located in capillaries, corresponding to blood stages of the parasites (Fig. 3). The number of detected signals was overall low, with most organs showing less than 50 signals per 10 fields of view (FOV) at low microscopic magnification (100x) (Table 2). Signals were primarily observed in the lungs, liver, spleen, and kidneys, but in some individuals, signals were also seen in the brain, gastrointestinal tract, heart and skeletal muscle, and skin. When examining corresponding locations of detected signals in adjacent HE-stained sections, no parasite structures were recognized, ruling out larger exo-erythrocytic stages (meronts) as possible source for the signal. Comparing the RNAscope results with the chromogenic ISH results, the form and distribution of signals was similar, as chromogenic ISH blood stage signals were also roundish and located in infected erythrocytes (Fig. 3E). However, in contrast to RNAscope, chromogenic ISH revealed parasites in only three of seven infected birds from the winter group (Additional file 2). Also, by chromogenic ISH, the abundance of signals was even lower with often only single signals detected in the lungs, liver, spleen, and kidneys, while other organs remained negative. Neither by RNAscope, nor by chromogenic ISH, signals were located in tissue cells, providing no evidence for exo-erythrocytic stages persisting in the organs from birds dissected during winter.

**Table 2:** *Plasmodium relictum* pSGS1 blood stages detected by RNAscope in situ hybridization (ISH) in experimentally infected *Serinus canaria* examined during winter (blood film-latent infection, *n* = 7) and after relapse (*n* = 6)

Bird ID	Group	RNAscope ISH results^a, b, c^																
		he	lu	li	sp	ki	br	mu	sto	int	tr	oe	thm	thr	ski	bm	ova	tes
AH2203	Winter	+	+	+	+	+	+	+	+	−	+	−	−	N/A	−	−	N/A	N/A
AH2204	Winter	+	+	+	+	+	−	+	+	−	−	−	N/A	−	+	−	N/A	N/A
AH2205	Winter	−	+	+	+	+	−	+	−	−	−	−	−	−	−	−	N/A	N/A
AH2206	Winter	+	+ +	+	+	+	+	+	+	−	−	+	+	−	−	−	N/A	N/A
AH2207	Winter	+	+	−	+	+	−	−	−	−	−	−	−	−	−	−	−	N/A
AH2208	Winter	−	+	−	−	−	−	+	−	−	−	−	N/A	N/A	−	−	N/A	−
AH2209	Winter	−	−	−	−	−	−	−	−	−	−	−	N/A	N/A	−	−	N/A	N/A
AH2213	Relapse	+ +	+ + +	+ +	+ +	+	+	+ + +	+	N/A	+	+	−	N/A	+	−	+	N/A
AH2214	Relapse	+ + +	+ + +	+ + +	N/A	+ + +	+ + +	+ + +	+ +	+ + +	+	+ + +	N/A	N/A	+ + +	+ + +	N/A	+ + +
AH2215	Relapse	+	+ + +	+ + +	+ + +	+	+	+	+	+	N/A	+	N/A	N/A	+	+	N/A	N/A
AH2216	Relapse	+	+	+	+	+	+	+	−	+	+	−	N/A	N/A	−	−	N/A	−
AH2217	Relapse	N/A	+	+	+	+	−	−	−	+	−	−	N/A	N/A	−	−	N/A	−
AH2218	Relapse	+	+ + +	+	+ + +	+ +	+	+	+	+	+	+	+	N/A	+	+	N/A	+

^a^*he* heart, *lu* lung, *li* liver, *sp* spleen, *ki* kidney, *br* brain, *mu* skeletal muscle, *sto* stomach, *int* intestines, *tr* trachea, *oe* oesophagus, *thm* thymus, *thr* thyroid, *ski* skin, *bm* bone marrow, *ova* ovary, *tes* testicle

^b^RNAscope ISH signals found per 10 fields of view (FOV) at 100 × microscopic magnification: low grade ‘ + ’: 1–50; moderate grade ‘ +  + ’: 51–100; high grade ‘ +  +  + ’: > 100; negative '-': no signals detected

^c^*N/A* not available

In the birds from the relapse group, both the RNAscope ISH and chromogenic ISH revealed signals in all infected individuals, showing blood stage signals in diverse organs (Table 2 and Additional file 2). The detected signals were similar to those observed in the winter group with regard to form, size and location (Fig. 3). However, compared to the birds investigated during winter, the abundance of RNAscope ISH signals found in the birds from the relapse group was higher, with four of six infected individuals showing more than 100 blood stage signals/10 FOV in several of their organs (Table 2). Signals were also observed in more organs compared to the winter group, including lungs, liver, spleen, kidneys, gonads, brain, gastrointestinal tract, heart, and skeletal muscle, skin, and bone marrow. Similar to the results from the winter group, chromogenic ISH generally revealed fewer blood stage signals in the relapse group as compared to RNAscope ISH (Additional file 2). No evidence for exo-erythrocytic tissue stages was found in the birds from the relapse group by RNAscope ISH or chromogenic ISH. All uninfected control birds were negative by in situ hybridizations.

## Discussion

Relapses of parasitaemia are a common phenomenon in birds infected with avian malaria or other haemosporidian parasites, indicating that many birds do not eliminate the parasites entirely but stay chronically infected for extended periods of time [1, 14, 15, 17]. However, the mechanisms of parasite persistence in the avian host are not well understood due to little available information and the lack of targeted experimental research including investigation of tissues during latent infections. This study presented an attempt to explore persistence of *P. relictum* pSGS1 parasites in the tissues of experimentally infected canaries during the latent phase of infection using a novel, highly sensitive RNAscope ISH approach. Based on the hypothesis that persisting exo-erythrocytic stages give rise to parasitaemia relapses in spring, the primary goal was to find putative dormant tissue stages in organs of chronically infected birds during winter. Despite systematic examination of diverse tissues and organs, no tissue stages were found in birds with latent infections, suggesting absence of exo-erythrocytic dormant stages in *P. relictum* pSGS1 blood-inoculated canaries. Different reasons might explain this result.

First, failure to detect possible persistent or dormant stages might be related to methodological issues. Technically, RNAscope ISH allows single-molecule detection, which is achieved by employing a set of multiple double “Z” probes (usually ~ 20 probe pairs) and a signal amplification system [52]. However, due to difficulties in designing multiple probe pairs targeting the *18S* ribosomal RNA of *P. relictum* pSGS1, the custom-made RNAscope probe consisted of only two probe pairs, hence providing less targets for the signal amplification step. While the probe reliably identified *P. relictum* pSGS1 stages during active infection and showed superior sensitivity compared to conventional CISH—demonstrated by higher numbers of detected signals—it might not be sensitive enough for detecting quiescent developmental stages with extremely downregulated or even no ribosomal activity. In such cases, it might be advantageous to target genomic DNA (e. g. ribosomal DNA) instead of RNA, however whether single copies of genes provide sufficient targets needs to be tested in future. In addition to technical issues related to the RNAscope probe, persistent tissue stages might have been missed, as only few sections of each organ were examined. Assuming that dormant stages are scarce, this possibility cannot be ruled out and certainly presents one of the main limitations of this study. Unfortunately, due to high costs of the RNAscope ISH assay, only a limited number of sections could be processed in this study, preventing a more thorough investigation of organs, which might be necessary to locate rare parasite stages. The fact, that there are no cues on where in the avian host dormant stages might persist, further complicates focused in-depth examination of specific tissues or organs, highlighting difficulties in the search for persisting tissue stages of avian malaria or other haemosporidian parasites, as was the case in mammalian malaria species [29].

Besides methodological reasons, an alternative explanation for the failure to detect persisting tissue stages is that dormant exo-erythrocytic stages were not formed in blood-inoculated canaries using this *P. relictum* pSGS1 strain, ruling out presumed dormant tissue stages as responsible cause for the observed recurrent parasitaemia. In fact, the results of this study support this conclusion. While phanerozoites have been implicated in the persistence of avian malaria infections, information about these developmental stages on the lineage level is limited [11, 45]. In this study, pSGS1 exo-erythrocytic stages were not found, neither during latency in winter, nor during recurrent parasitaemia in spring. Moreover, the bird which died early during the experiment (84 dpi), showed no exo-erythrocytic stages, suggesting that the used strain of *P. relictum* pSGS1 does not develop phanerozoites which could give rise to parasitaemia relapse. This result tempts to speculate whether the lack of phanerozoite development presents a fixed evolutionary trait of this lineage or reflects loss of the ability of the used pSGS1 strain to infect tissue cells due to multiple blood passages in laboratory birds.

The findings of this study do not allow generalizations about the absence or presence of phanerozoites in related lineages or even species because the development might differ between strains [45]. Similarly, the results do not rule out the existence of dormant exo-erythrocytic stages of avian *Plasmodium* spp. during natural infections. Considering that human malaria parasites develop hypnozoites from liver-cell invading sporozoites early during infection [37], it is conceivable, that avian malaria parasites form similar dormant stages after inoculation with sporozoites. To test this hypothesis, future experimental infection studies using sporozoite inoculation are necessary.

Regardless of speculated reasons, the absence of dormant or persisting tissue stages in the birds investigated in this study calls for alternative explanations for the observed parasitaemia relapses and persistence of infections. First, it should be highlighted, that parasitaemia relapses were observed in at least five of six birds in spring, demonstrating the ability of the used pSGS1 strain to cause recurrent parasitaemia in chronically infected canaries maintained in laboratory conditions. Hence, it is plausible to assume that most birds dissected during winter would also have experienced recurrent parasitaemia, ruling out clearance of infection as ultimate reason the failure to detect persisting exo-erythrocytic stages in these individuals. Importantly, RNAscope ISH revealed few blood stage signals in the tissues of the birds, demonstrating that the parasites were not entirely cleared from the blood, which is consistent with earlier reports of transient parasitaemia in birds infected with other lineages of *P. relictum* [45]. However, the number of blood stages detected by ISH was much lower in birds from the winter group compared to birds examined after re-appearance of parasitaemia, indicating replication of the parasites during early spring. This is supported by the observation of erythrocytic meronts in blood films during relapse. Considering, that no exo-erythrocytic stages were found during latent infections, it is concluded, that the observed recurrence of parasitaemia in spring was caused by erythrocytic merogony of the parasites and hence reflects recrudescence rather than true relapse involving tissue stages. Along with the observation of long-lasting parasitaemia throughout December (several months after inoculation), it seems that chronic infections were sustained through persistence of the parasites in the blood circulation. Given the average lifespan of a month of healthy avian erythrocytes [55], it is concluded that parasite persistence throughout winter months was likely mediated by continuous, but reduced erythrocytic merogony. This is supported by the observation of a few erythrocytic meronts in the peripheral blood of infected birds in December. Whether these findings can be translated to free-living birds experiencing natural winter conditions remains to be investigated. From an epidemiological point of view, decreasing erythrocytic merogony to a minimum in times when vectors for transmission are absent seems reasonable, also to avoid additional pressure on avian hosts already experiencing greater environmental stress during cold times [56]. However, it raises the question about possible mechanisms responsible for reduced merogony at such low levels. While host immunity probably plays a substantial role in limiting parasite numbers it is also possible to speculate that parasite-related factors are implicated in controlling multiplication rates by decelerating asexual replication, for example via prolonged cycle duration or reduced production of merozoites. In vitro studies with human and rodent *Plasmodium* parasites have shown some adaptive variations in regarding cell cycle durations and replication rates over time [57, 58], but whether avian *Plasmodium* exhibit similar phenotypic plasticity that could explain low parasitaemia levels in birds is hypothetical.

The result that in birds with latent infections parasite blood stages were detected by ISH but not in blood films could reflect varying sensitivity of different diagnostic protocols. Extended microscopic screening of blood films obtained during winter might reveal parasite stages, but this was not tested during this study. Alternatively, the contrasting results might also relate to different detection probabilities when examining blood samples obtained from the peripheral circulation or blood-containing tissue samples. In this context, it is interesting to note, that during winter, blood stages were mostly located in capillaries of organs, whereas in birds examined during spring, ISH revealed plenty blood stages not only in the capillaries but also larger vessels. These findings suggest, that reduced parasitaemia during winter might be accompanied by sequestration of parasitized erythrocytes in the microcirculation, which could present a strategy of the parasites to evade immunity and minimize splenic clearance, as has been reported for some human and rodent malaria parasites [59–62]. Accumulation of avian *Plasmodium* stages in certain tissues (e. g. bone marrow, fat, serous membranes of internal organs) has been reported previously [11, 14, 51, 61, 63], but not yet investigated systematically. Unfortunately, the overall low number of ISH-detected blood stages during winter and a missing comparison of blood stage distribution in tissues of birds exhibiting extremely low parasitaemia during summer, do not allow for final conclusions about tissue sequestration of *P. relictum*-infected erythrocytes during latent infections. Future studies are needed to explore tissue sequestration during avian malaria, which might not only help to understand persistence of infections but have also implications for ecological studies examining temporal variation of parasite prevalence.

## Conclusions

This study demonstrates persistence of *P. relictum* pSGS1 parasites in the microcirculation of chronically infected canaries during apparent latency, suggesting sequestration of infected blood cells during winter. In absence of detected exo-erythrocytic tissue stages, persistence is likely mediated by continuous low-level erythrocytic merogony throughout the year and re-appearance of parasitaemia the result of increased erythrocytic merogony, therefore representing recrudescence and not relapse in blood-induced infections. The latter experimental infections are easy to design; they predominate in current experimental avian malariology research but might be unsuitable for the search of dormant exo-erythrocytic stages. Further experimental studies, preferably sporozoite-induced infections, are needed to explore the role of tissue sequestration and possible dormant exo-erythrocytic stages in long-term persistence of avian malaria and related haemosporidian infections. The superior sensitivity of the RNAscope ISH technology might be advantageous for histological investigations approaching these research questions.

### Supplementary Information

**Additional file 1. **Kidney sections of an infected bird tested with the RNAscope in situ hybridization (ISH) assay for detecting *Plasmodium relictum* pSGS1 stages. (**A**) RNAscope ISH showed bright red labelling of *P. relictum* pSGS1 stages, while mild unspecific background staining was observed in some renal tubuli (right side of the image). (**B**) Similar background staining of renal tubuli (right side of the image) was observed when the RNAscope probe was omitted during the assay. Scales = 20 µm.

**Additional file 2.** *Plasmodium relictum* pSGS1 blood stages detected by chromogenic in situ hybridization (CISH) in experimentally infected *Serinus canaria* examined during winter (blood film-latent infection, n = 7) and after relapse (n = 6).

## Data Availability

All data generated or analysed during this study are included in this published article and its supplementary information files.

## Abbreviations

ISH: In situ hybridization
dpi: Days post infection
*cytb*: Cytochrome b
FFPE: Formalin-fixed paraffin-embedded
HE: Haematoxylin–eosin
SSC: Saline-sodium citrate
BCIP: 5-Bromo-4-chloro-3-indolyl phosphate
NBT: Nitro-blue tetrazolium chloride
