# Supplementary figures and images for: RNAscope in situ hybridization reveals microvascular sequestration of Plasmodium relictum pSGS1 blood stages but absence of exo-erythrocytic dormant stages during latent infection of Serinus canaria
Source: Malar J. 2024 Mar 8;23:70. doi: 10.1186/s12936-024-04899-x (PMC10924391; doi:10.1186/s12936-024-04899-x)

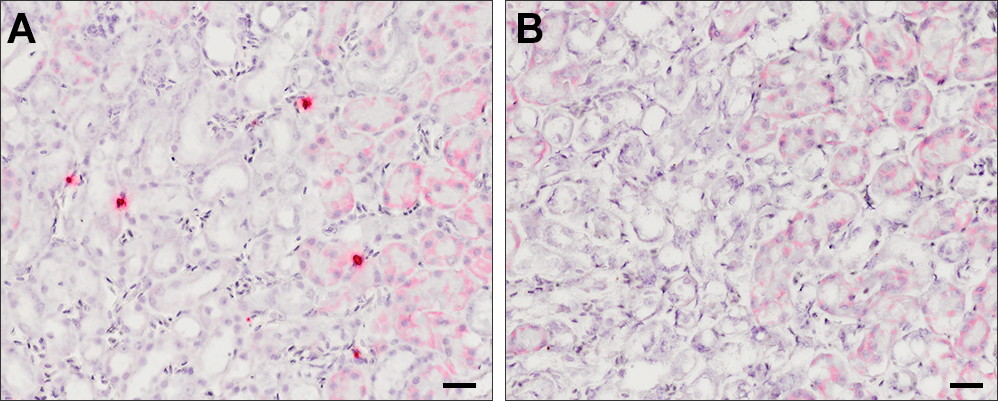

Supplement: Supplementary file 1 — Additional file 1. Kidney sections of an infected bird tested with the RNAscope in situ hybridization (ISH) assay for detecting Plasmodium relictum pSGS1 stages. (A) RNAscope ISH showed bright red labelling of P. relictum pSGS1 stages, while mild unspecific background staining was observed in some renal tubuli (right side of the image). (B) Similar background staining of renal tubuli (right side of the image) was observed when the RNAscope probe was omitted during the assay. Scales = 20 µm. [file 12936_2024_4899_MOESM1_ESM.jpg]
